# Association Study of Single Nucleotide Polymorphisms of Endoplasmic Reticulum Aminopeptidase 1 and 2 Genes in Iranian Women with Preeclampsia

**Published:** 2019-03

**Authors:** Hamid DARGAHI, Mohammad Hossein NICKNAM, Mahroo MIRAHMADIAN, Mahdi MAHMOUDI, Saeed ASLANI, Maryam SADROSADAT, Robabeh GHODSSI-GHASSEMABADI, Ali Akbar AMIRZARGAR

**Affiliations:** 1. Department of Medical Immunology, School of Medicine, Tehran University of Medical Sciences, Tehran, Iran; 2. Molecular Immunology Research Center, Tehran University of Medical Sciences, Tehran, Iran; 3. Rheumatology Research Center, Tehran University of Medical Sciences, Tehran, Iran

**Keywords:** Endoplasmic reticulum aminopeptidases 1 and 2, Preeclampsia, Single nucleotide polymorphism

## Abstract

**Background:**

Endoplasmic reticulum aminopeptidases 1 and 2 (ERAP1 and 2) are involved in blood pressure regulation and single nucleotide polymorphisms (SNPs) of these genes have been linked to preeclampsia. This study intended to assess the association of *ERAP1* and *2* genes polymorphism with Iranian preeclamptic women.

**Methods:**

In this case-control study, 148 preeclamptic and 133 pregnant women were selected from the Kosar Hospital, Qazvin, Iran, during 2013–2015. In order to genotype the subjects for rs28096, rs30187, rs26653, rs3734016, rs34750 and rs2549782, rs17408150 for *ERAP1* and *2* genes, respectively, Real-Time PCR allelic discrimination approach was exploited.

**Results:**

Neither allelic nor genotype frequencies of all seven polymorphisms were significantly different between two groups. Though, ACGACTT and GTCAGGA haplotypes were related with decreased (*P*=0.0079, OR=0.559, 95% CI: 0.363–0.861 and *P*=0.02, OR=0.417, 95% CI: 0.194–0.896, respectively), but ACGACGT and GTGACTT haplotypes were associated with an increased (*P*=0.00082, OR=3.657, 95% CI: 1.630–8.206 and *P*=0.02, OR=2.401, 95% CI: 1.119–5.151, respectively) risk of preeclampsia. Moreover, some positions were detected to be in linkage disequilibrium.

**Conclusion:**

Ongoing investigation resulted differently from before performed studies considering the role of *ERAP1* and *ERAP2* gene polymorphisms in predisposing women to preeclampsia, emphasizing on the genetic structure differences among various racial populations.

## Introduction

Preeclampsia (PE) is a serious pregnancy-related disorder, diagnosed by the simultaneous increased blood pressure and proteinuria, which affects 3%–5% of pregnancies according to most population-based studies. Invariably, the two hallmarks to distinguish the preeclampsia have been credited to be recent onset of hypertension and proteinuria during pregnancy (1). The patho-physiology of PE is much more than the increased blood pressure and altered renal function.

Decreased perfusion to almost all organs, impaired coagulation cascade, and decreased plasma volume are also the further potential complications of preeclampsia (2). The role of genetics, documented with the observation of both maternal and paternal genetics involvement in contributing to preeclampsia susceptibility, demonstrated through extensive epidemiological studies (3, 4).

The endoplasmic reticulum aminopeptidases 1 and 2 (ERAP1 and 2), which play important roles in the immune system, are involved in the antigen presentation via MHC molecules and found within the endoplasmic reticulum (5). Moreover, ERAP1 and 2 are involved in blood pressure regulation by playing a role in the reninangiotensin-aldosterone pathway. *In vitro* investigations have demonstrated that the ERAP1 enzyme is efficiently involved in cleaving and inactivation of angiotensin II, plus its converting potential of kallidin to bradykinin (6). ERAP2 enzyme can process the angiotensin III to cleave it to angiotensin IV. Moreover, this enzyme can convert kallidin to bradykinin and have no effect in cleaving vasopressin, angiotensin II, and oxytocin (7).

PE predisposition has been reported in association with distinct single nucleotide polymorphisms (SNPs) in *ERAP2* gene in various ethnical populations (8, 9). The minor allele of rs2549782 (G) causes the substitution of a nonconservative amino acid (N392K). This variation changes ERAP2 enzyme activity through impressing the substrate specificity of the active site of enzyme (10).

Considering the role of ERAP1 and 2 enzymes in blood pressure regulation and regarding well-established manifestation of preeclampsia, impaired blood pressure regulation, this study was conducted to evaluate whether SNPs of *ERAP1* and *ERAP2* genes were associated with PE susceptibility in Iranian women.

## Methods

### Subjects

In this case-control study, genetic analysis was performed on a total of 281 individuals, which comprised of 148 unrelated preeclamptic women, whose diagnosis had been confirmed based upon American College of Obstetricians and Gynecologists (ACOG) criteria (11) and 133 healthy pregnant women with no history of autoimmune disease or preeclampsia susceptible condition (e.g. kidney dysfunctions, diabetes, twining delivery). The diagnosis of preeclampsia was confirmed clinically and paraclinical by means of qualified gynecologists. The patients were selected from Kosar Hospital, Qazvin, Iran during 2013–2015.

Table 1 depicts the clinical specifications of study subjects and their values as Mean ± standard deviation (SD).

**Table 1:** Clinical specifications of the study subjects

***Participant Characteristics***	***Patients (n=148)***	***Controls (n=133)***	***P value***
Age(yr)	32.7 ± 6.9	33.9 ± 5.3	0.18
Diastolic Blood Pressure (mm Hg)	87.3 ± 12.1	77.9 ± 5.73	<0.0001
Systolic Blood Pressure (mm Hg)	138.8 ± 21.55	117 ± 7.6	<0.0001
Proteinuria	1.95 ± 1.2	0.13 ± 0.09	<0.0001
Abortion	13 (Yes)	10 (Yes)	0.07
	135 (No)	123 (No)	
Time of Abortion	0.15 ± 1.2	0.27 ± 0.61	0.33
Height (cm)	164.2 ± 6.9	161.2 ± 7.75	0.0007
Weight (kg)	78.9 ± 11.5	69.8 ± 13.11	0.01
BMI (kg/m^2^)	29.28 ± 0.31	26.87 ± 0.39	<0.0001

Ethical committee of Tehran University of Medical Sciences, Tehran, Iran corroborated the study and informed written consents were signed by all patients and controls. Moreover, the study followed the ethical standards of the Helsinki Declaration.

Five ml of venous blood was taken from each patient and healthy pregnant woman through EDTA-anticoagulated Venoject Tubes. Genomic DNA was extracted from whole blood using standard procedures of Salting-Out method as described previously (12). The concentration and purity of the extracted DNA were determined by spectrophotometry.

## Positional candidate SNP selection and Real-Time PCR genotyping

All the samples were genotyped for rs28096, rs30187, rs26653, rs3734016, rs34750 and rs2549782, rs17408150 for *ERAP1* and *2* genes, respectively, using Real-Time allelic discrimination Taq-Man assays (Applied Biosystems, Foster City, USA). Table 2 displays chosen SNPs and their details.

**Table 2:** Properties of investigated polymorphisms in preeclampsia patients and healthy controls

***Gene***	***SNP ID***	***Minor Allele***	***Variation***	***Position***	***Amino Acid Change***
*ERAP-1*	rs28096	A	A/G	96135000	Intronic
	rs30187	T	C/T	96150086	Lys528Arg
	rs26653	C	C/G	96165006	Arg127Pro
	rs3734016	G	A/G	96165220	Glu56Lys
ERAP-2	rs34750	G	C/G	96168562	Intronic
	rs2549782	G	G/T	96256756	Lys392Asn
	rs17408150	A	A/T	96265014	Leu669Gln

Briefly, all PCR reactions mixture contained approximately 25–75 ng of DNA, 5 μl Taq-Man Master Mix containing Taq DNA polymerase and dNTPs (Applied Biosystems, Foster City, USA), 0.25μl Taq-Man Genotyping Assay mix containing primers and FAM or VIC labeled probes (Applied Biosystems, Foster City, USA), and distilled water for a final volume of 10 μl. Real-Time allelic discrimination PCR was performed by StepOnePlus Real-Time PCR system (Applied Biosystems, Foster City, USA). The Real-Time PCR conditions were: initially 60 °C for 30 sec and then 95 °C for 10 min, and subsequently 40 cycles of amplification (95 °C for 15 sec and 60 °C for 1 min), and finally 60 °C for 30 sec.

### Statistical analysis

Genotype and allelic distribution between case and control groups were implemented by Chi-Square (χ^2^) test. Pearson’s χ^2^-tests were applied to test for significant differences of both genotype and allele frequencies between two groups. Universally, α=0.05 was regarded as the significant level. All probability values were calculated from two-tailed test. Moreover, the odds ratio (OR) and 95% confidence interval (CI) were calculated. The genotype distributions of chosen SNPs were tested for deviation from Hardy-Weinberg equilibrium (HWE) in case and control. The Bonferroni correction approach was exerted in multiple statistical testing (i.e. *P*-value<0.01) to recognize statistically significant results, adjusting the multiple comparisons, and controlling the false discovery rate (FDR) (13). Moreover, several parts of statistical analysis were performed using the SPSS for Windows (ver. 22.0, IBM SPSS Inc., USA). Additionally, the SHEsis online tool was exerted for analyzing the haplotype and genotype, and also Hardy-Weinberg equilibrium for gene-gene interactions (14).

## Results

### Clinical Data

Specification of preeclamptic women and control group are barely shown through Table 1. Case subjects with the mean age of 32.7±6.9 were found to be age-matched with healthy control group with that of 33.9 ± 5.3 (*P*>0.05). To get more equitable comparison, several clinical conditions impressing PE, diastolic blood pressure (*P*<0.0001), systolic blood pressure (*P*<0.0001), proteinuria (*P*<0.0001), height (*P*=0.0007), weight (*P*=0.01) and Body Mass Index (BMI) (*P*<0.0001) were significantly different between two categorized study subjects. Preeclamptic women, as could have expected, suffered from both high blood pressure (systolic and diastolic) and proteinuria which were both significantly high in comparison to control group. But to our wonder, abortion (*P*=0.07) and its times (*P*=0.33) depicted no significant difference between cases and control groups.

### Alleles and Genotypes Frequencies

Allelic and genotype frequencies in of the SNPs in *ERAP1* and *ERAP2* genes in preeclamptic patients and controls are presented in Table 3.

**Table 3:** Allele and genotype distribution of various SNPs of *ERAP1, 2* in preeclampsia cases and healthy controls

***dbSNP***	***Alleles/Genotypes***	***Case (n=148) N (%)***	***Control (n=133) N (%)***	***P value***	***OR (95% CI)***
rs28096	A	110 (37.4)	105 (39.5)	0.61	0.91 (0.65–1.28)
	G	184 (62.16)	161 (60.5)	0.61	1.09 (0.77–1.53)
	AA	19 (12.9)	20 (15.0)	0.61	0.83 (0.42–1.65)
	AG	72 (49.0)	65 (48.9)	0.98	1.00 (0.62–1.60)
	GG	56 (38.1)	48 (36.1)	0.72	1.08 (0.67–1.77)
HWE			0.798		
rs30187	T	129 (43.6)	102 (38.9)	0.26	1.21 (0.86–1.69)
	C	167 (56.4)	160 (61.1)	0.26	0.82 (0.58–1.15)
	TT	28 (18.9)	18 (13.7)	0.24	1.46 (0.76–2.79)
	TC	73 (49.3)	66 (50.4)	0.86	0.95 (0.59–1.53)
	CC	47 (31.8)	47 (35.9)	0.46	0.83 (0.50–1.36)
HWE			0.498		
rs26653	C	122 (41.5)	112 (42.1)	0.88	0.97 (0.69–1.36)
	G	172 (58.5)	154 (57.9)	0.88	1.02 (0.73–1.43)
	CC	27 (18.4)	25 (18.8)	0.92	0.97 (0.53–1.77)
	CG	68 (46.3)	62 (46.6)	0.95	0.98 (0.61–1.57)
	GG	52 (35.4)	46 (34.6)	0.89	1.03 (0.63–1.69)
HWE			0.618		
rs3734016	A	293 (99.0)	266 (100)	0.099	-
	G	3 (1)	0 (0)	0.099	-
	AA	145 (98.0)	133 (100)	0.099	-
	AG	3 (2)	0 (0)	0.099	-
	GG	0 (.0)	0 (0)	-	-
HWE			1.00		
rs34750	C	174 (59.18)	155 (58.27)	0.82	1.03 (0.74–1.45)
	G	120 (40.82)	111 (41.73)	0.82	0.96 (0.68–1.34)
	CC	54 (36.73)	47 (35.34)	0.80	1.06 (0.65–1.73)
	CG	66 (44.90)	61 (45.86)	0.87	0.96 (0.60–1.54)
	GG	27 (18.37)	25 (18.80)	0.21	0.97 (0.53–1.77)
HWE			0.51		
rs2549782	T	121 (40.88)	107 (40.23)	0.87	1.02 (0.73–1.43)
	G	175 (59.12)	159 (59.77)	0.87	0.97 (0.69–1.36)
	TT	51 (34.5)	43 (32.3)	0.70	1.10 (0.66–1.80)
	TG	73 (49.3)	73 (54.9)	0.35	0.80 (0.50–1.27)
	GG	24 (16.2)	17 (12.8)	0.41	1.32 (0.67–2.58)
HWE			0.10		
rs17408150	A	25 (8.4)	34 (12.8)	0.094	0.62 (0.36–1.08)
	T	271 (91.6)	232 (87.2)	0.094	1.58 (0.92–2.74)
	AA	0 (0)	3 (2.3)	0.066	-
	AT	25 (16.9)	28(21.1)	0.37	0.76 (0.41–1.38)
	TT	123 (83.1)	102(76.7)	0.17	1.49 (0.83–2.69)
HWE			0.52		

OR, odds ratio; CI, confidence interval; - means Not Calculated

Distributions of all seven polymorphisms in healthy group revealed no evidence of deviation from HWE.

#### ERAP1 Polymorphisms

In rs28096 SNP, PE patients exhibited 37.4% A and 62.6% G alleles, which had similar frequencies in the control women. Both alleles of this SNP did not disclose statistically significant prevalence between two groups (*P*=0.61). The AG genotype was identified in 49% of the patients and 48.9% of the heathy control women (*P*=0.98, OR=1.004, 95% CI= 0.62–1.60). The AA genotype was more frequently distributed in the control group compared with the patient group, however, this distribution difference was not statistically significant (15% *vs*. 12.9%, respectively; *P*=0.61, OR=0.83, 95% CI= 0.42–1.65). As well, with regard to the *ERAP1* rs28096 polymorphism, the GG genotype did not disclose statistically significant prevalence between the PE and healthy women (38.1% *vs*. 36.1%, respectively; *P*=0.72, OR=1.08, 95% CI= 0.67–1.77).

Variations of the *ERAP1* gene rs30187 SNP, the T and C alleles, demonstrated somewhat equal frequencies between PE patients and healthy controls (43.6% *vs*. 38.9% for T allele; 56.4% *vs*. 61.1% for C allele); hence, the frequency differences between the two groups was not statistically significant (*P*=0.26). Among the three genotypes, the TC genotype had the highest frequency in both PE and healthy women (49.3% *vs*. 50.4%, respectively), but insignificantly distributed between PE patients and control women (*P*=0.86, OR=0.95, 95% CI= 0.59–1.53). On the contrary, the TT genotype represented the least frequency in both studied groups (18.9% *vs*. 13.7%) and no statistically significant difference was seen in the genotype frequency between PE patients and healthy subjects (*P*=0.24, OR=1.46, 95% CI= 0.76–2.79). Among the studied individuals, 31.8% of PE women and 35.9% of controls showed CC genotype; hence, no statistically significant difference was detected in frequency of this genotype between the PE and control women (*P*=0.46, OR=0.83, 95% CI= 0.50–1.36).

In rs26653 polymorphism, while G allele was almost highly found over than C alleles, but the frequencies of both alleles were significantly different neither in preeclamptic patients nor healthy pregnant women. Three genotypes of the variation, additionally, were failed to show significant difference in regards frequency distribution between case and control groups.

In *ERAP1* gene rs3734016 SNP, PE patients and control women had 99% and 100% A allele frequency. On the contrary, the G allele was observed less frequently in both groups (1% *vs*. 0%). Both alleles did not indicate significantly different frequency between PE and healthy women (*P*=0.09). While the AA genotype was approximately seen in all the PE and healthy pregnant women (98% *vs*. 100%), none of the study subjects had the GG genotype. The frequency of three genotypes of *ERAP1* gene rs3734016 SNP did not differ significantly between PE patients and healthy women.

Both C and G alleles of rs34750 polymorphism were equally distributed, even though C and G alleles were detected in 59.1% and 40.8% of PE women, almost similar to 58.2% and 41.7% of control group. As a result, neither C nor G allele showed significant difference between patients and control women (*P*=082).

#### ERAP2 Polymorphisms

In the rs2549782 SNP, T and G alleles were found in 40.8% and 59.1% patients and 40.2% 59.7% controls; due to equal frequency between groups for both, no significant difference was observed (*P*=0.87). The TG genotype was seen in 49.3% and 54.9% of the PE and healthy pregnant women, despite slightly more common genotype, had no significant difference between patients and controls (*P*=0.35, OR=0.80, 95% CI= 0.50–1.27). Meanwhile, both TT and GG could not be represented significantly between two groups (*P*=0.70, OR=1.10, 95% CI= 0.66–1.80 and *P*=0.41, OR=1.32, 95% CI= 0.67–2.58, respectively).

The A allele of *ERAP2* gene rs17408150 polymorphism was less found in 8.4% of patients and closely in 12.8% of healthy women, resulting in insignificant difference between PE patients and control group (*P*=0.09). Albeit being most common allele, T was found nearly same in percentage of 91.6 and 87.2 in PE and healthy women, respectively, and frequency difference was insignificant (*P*=0.09). The TT genotype for abovementioned SNP was highly but similarly observed in either PE or healthy women (83.1% *vs*. 76.7%, respectively), resulting in no statistically significant difference in prevalence between two studied groups (*P*=0.17, OR=1.49, 95% CI= 0.83–2.69). The AA genotype was never seen in patients but in 2.3% of controls, however, with no significant difference (*P*=0.06). The AT geno-type did not show significantly different prevalence between the two studied groups (*P*=0.37, OR=0.76, 95% CI= 0.41–1.38).

#### Haplotype Frequencies

Due to their adjacent location on 5q15 and since their supposed derivation from a shared ancestral gene (7), *ERAP1* and *ERAP2* haplotypes were performed simultaneously. Here, we employed SHEsis online tool to measure the haplotype frequencies between cases and controls. The ACGACTT haplotype was the most frequent haplo-type of control group and significantly differed to that of preeclamptic women (23% *vs*. 15%, respectively; *P*=0.007918, OR=0.55, 95% CI= 0.36–0.86). This haplotype was found to be associated with decreased risk of PE in investigated population. Furthermore, the frequencies of ACGACGT and GTGACTT haplotypes were detected to have statistically significant differences in the patients and, therefore, were contributing factors for increased risk of PE as susceptibility haplotypes (10% *vs*. 3%; *P*=0.00082, OR=3.65, 95% CI= 1.63–8.20 and 8.5% *vs*. 4%; *P*=0.02, OR=2.40, 95% CI= 1.12–5.15, respectively). On the other side, the GTCAGGA haplotype was more frequent in control group compared to PE patients and attributed to protective haplotype (8% *vs*. 3.6%, respectively, *P*=0.02, OR=0.41, 95% CI= 0.19–0.89).

#### Linkage Disequilibrium Tests

In order to evaluate the linkage disequilibrium (LD) tests of *ERAP1* and *ERAP2* polymorphisms, the SHEsis online software was used. *ERAP1* gene rs28096 polymorphism was seen to be in LD with rs30187 and rs3734016 SNPs for *ERAP1* and rs17408150 SNP for *ERAP2* according to D′ values of 0.94, 0.98, and 0.89, respectively, but not based on *r*^*2*^ values. Moreover, rs30187 polymorphism of *ERAP1* was in LD with rs3734016 SNP of *ERAP1* and rs17408150 of *ERAP2* (D′=0.81 and 0.95, respectively), but *r*^*2*^ values were incongruent.

As more striking value, rs26653 SNP was detected to be in LD with rs34750 SNP, both placed in *ERAP1* gene with worthy values of both D′=0.99 and *r*^*2*^= 0.97. The mentioned position was in LD with rs3734016 polymorphism.

**Fig. 1:**
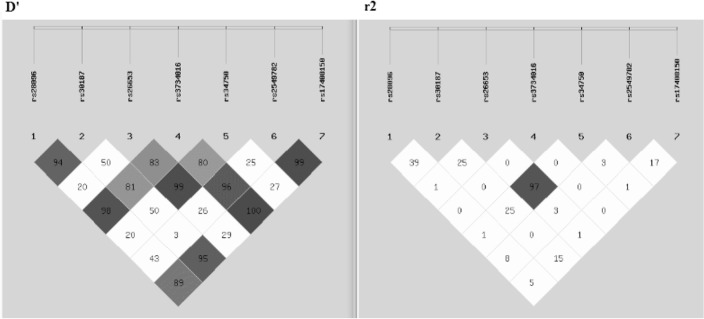
Linkage disequilibrium (LD) diagrams for *ERAP1* and *2* genes in Iranian preeclamptic patients. Diagrams are for seven SNPs in LD values (SNP order: rs28096, rs30187, rs26653, rs3734016, rs3734016, rs34750, rs2549782, rs17408150). Left and right diagram demonstrate the D′ and *r^2^* values, respectively. Dark blocks represent the values between 0.85 and 1.00, grey blocks between 0.50 and 0.85, and white blocks show less than 0.50 values

The rs3734016 SNP for *ERAP1* gene was observed to be in LD with rs2549782 polymorphism of *ERAP2* gene. On the other side, these two genes depicted LD in terms of rs3734016 and rs17408150 with D′ value of 1.00. Two positions of *ERAP1* gene, rs2549782 and rs17408150 were in LD according to D′=0.99 but not *r*^*2*^= 0.17 (Fig. 1).

## Discussion

This study attempted to disclose the possible implications of the *ERAP1* and *ERAP2* gene polymorphisms in predisposing the pregnant women to preeclampsia using Real-Time allelic discrimination Taq-Man assays. We observed that allele and genotype frequencies of rs28096, rs30187, rs26653, rs3734016, rs34750 and rs2549782, rs17408150 for *ERAP1* and *ERAP2* genes, respectively, were not associated with PE. However, four haplotypes of ACGACTT, GTCAGGA, ACGACGT, and GTGACTT were associated with the disease risk. Moreover, there was LD between the SNPs.

“Preeclampsia, a hypertensive condition of gestation, is characterized by a recent onset of hyper-tension and also a proteinuria after approximately 20 weeks of pregnancy” (15, 16). The genetic background of preeclampsia has been debated for long to date, although epidemiological data vigorously advocating both maternal and paternal contributions for being subject to PE (3, 4). Nonetheless, the heritable transmission pattern of preeclampsia risk within families implies the role of genetic background in the etiopathogenesis of PE, despite the remained uncertainties of identification of predisposing genes (17, 18).

A wide genetic association study of PE revealed that six genes, *IGF1*, *IL4R*, *IGF2R*, *GNB3*, *CSF1*, and *THBS4*, with maternal-fetal genotype interactions, were associated with PE. These findings establish a multigenic inheritance pattern in onset of PE (19).

Both ERAP1 and ERAP2 molecules have been postulated to have key roles in regulating the blood pressure by being involved in the reninangiotensin system. Chinese Hamster Ovary cells used in *in vitro* studies have stipulated a cleavage role to the ERAP1 enzyme, which inactivated angiotensin II, alongside with converting the kallidin to bradykinin (6, 20). On the other hand, through a similar pathway, the ERAP2 enzyme cleaved the angiotensin III to angiotensin IV and converted kallidin to bradykinin, but without any hydrolytic activity on oxytocin, angiotensin II, and vasopressin (7). Through a case/control cohort genetic study in Japanese population to interrogate previously known and also novel SNPs in *ERAP1* gene, a significant association between the rs30187 SNP and hypertension was disclosed (20). Furthermore, rs30187 SNP could have been able to decline the efficiency of ERAP1 enzyme by over 60% in cleaving the angiotensin II to angiotensin III and also about 70% decrement in the enzyme’s ability for converting kallidin into bradykinin (21). However, our results, being incompatible with antecedent findings, had no association with susceptibility or protectivity towards preeclampsia.

An association was identified between polymorphisms in the *ERAP2* gene and the risk of PE in patients from both Australian and Norwegian populations. An association between the rs2549782 SNP with PE in an Australian/New Zealand population was detected. Furthermore, the rs17408150 SNP of *ERAP2* gene was seen to have association with PE risk in patients from Norwegian (22). There were associations between the already recognized polymorphisms in *ERAP2* gene, namely rs2549782 and rs17408150 SNPs, and risk of PE in other cohorts with Chilean and African American ethnicities. While the G allele of rs2549782 SNP was observed to have association with PE susceptibility in the African American population, no association was identified in PE patients with Chilean ethnicity. On the other side, they indicated no association of rs17408150 SNP with PE predisposition in the patients with Chilean population. However, rs17408150 prevalence was not evaluated in association with PE risk in the African American population because of lack of the minor allele of this SNP in the mentioned population (23). In our investigation, not to be compatible with what others found in Australian/Norwegian and Chilean/African-American populations, the rs2549782 SNP and rs17408150 SNP were not associated with PE in Iranian women.

A genetic association was identified between the *ERAP1* gene rs3734016 and rs34750 SNPs as well the *ERAP2* gene rs2549782 SNP in the Australian/New Zealand patients with PE. In the Norwegian cohort, *ERAP1* gene rs34750 SNP and *ERAP2* gene rs17408150 SNP were in relation with PE. The *ERAP2* gene rs2549782 and rs17408150 SNPs were also significantly associated with PE in both studied populations after experiment-wide level corrections (22). None of the *ERAP1* gene 734016 and rs34750 SNPs could be associated with PE proneness in Iranian women. The distinction between the number of individuals recruited for genetic analysis in different studies and heterogeneity of the genetic makeup among the individuals can somehow justify the discrepancies observed in different studies. Moreover, the experimental approach differences in genotyping methods can be another potential cause of such incongruity.

Interplay among several mutations within a single gene on the same haplotype would result in “super-allele” which has an enormous influence on the manifested phenotype, notwithstanding uncommon frequency (24). Furthermore, haplotypes constructed from SNPs, regardless of being functional, can occasionally provide greater power than single-marker analyses in terms of genetic disease associations, due to the predecessor’s makeup captured in the haplotype distribution (25). A remarkable impression of *vascular endothelial growth factor* (*VEGF*) gene haplotypes regarding three clinically relevant SNPs (rs1570360, rs2010963, and rs699947), harbored by the promoter region of the *VEGF* gene, has been reported in the development of PE (26). Furthermore, *eNOS* haplotypes may be contributing to the initiation and perpetuation of hypertensive conditions in gestation (27).

Despite allele and genotype frequencies of all SNPs of *ERAP 1* and *2* genes, according to our data, were failed to be significantly different between two investigated subjects, several haplotypes were dissimilarly distributed. Albeit the ACGACGT and GTGACTT haplotypes were significantly frequent in the patients, the ACGACTT and GTCAGGA haplotypes were seen to be significantly less frequent in PE cases compared with control group (Table 4).

**Table 4:** Overall haplotype associations of the single-nucleotide polymorphisms according to Haploview

***Block 1 Haplotypes***								***Frequencies***			

***Row***	***rs28096***	***rs30187***	***rs26653***	***rs3734016***	***rs34750***	***rs2549782***	***rs17408150***	***Hap.freq (Case)***	***Hap.freq (Control)***	OR^*^ (95% CI)	***P value***
1	A	C	C	A	G	T	T	31 (10)	26 (10)	1.05 (0.60–1.81)	0.85
2	A	C	G	A	C	G	T	29 (10)	7 (3)	3.65 (1.63–8.20)	0.00082
3	A	C	G	A	C	T	T	43 (15)	61 (23)	0.55 (0.36–0.86)	0.00791
4	A	T	C	A	G	G	A	1 (0.5)	1 (0.4)	-	-
5	G	C	G	A	C	G	T	41 (14)	39 (15)	0.91 (0.56–1.46)	0.70
6	G	C	G	A	C	T	T	12 (4)	17 (6)	0.63 (0.30–1.33)	0.22
7	G	T	C	A	G	G	A	10 (3.6)	20 (8)	0.41 (0.19–0.89)	0.02
8	G	T	C	A	G	G	T	18 (6.2)	14 (5.5)	1.12 (0.55–2.30)	0.74
9	G	T	C	A	G	T	T	56 (19.3)	40 (15)	1.30 (0.83–2.03)	0.24
10	G	T	G	A	C	G	A	12 (4.4)	11 (4)	1.02 (0.44–2.32)	0.96
11	G	T	G	A	C	G	T	2 (0.8)	2 (1.1)	-	-
12	G	T	G	A	C	T	T	24 (8.5)	9 (4)	2.40 (1.12–5.15)	0.02

* OR, odds ratio; CI, 95% confidence interval for difference between Haplotype frequency of case-control; -means Not Calculated

In another point of view, several positions of the *ERAP1* gene were in linkage disequilibrium with *ERAP2* gene*. ERAP1* and 2 could collectively affect the risk of PE in this study group and even multifactorial polygenic basically play noticeable roll in development of PE.

## Conclusion

This study was the first study of its type to investigate the role of *ERAP1* and *ERAP2* gene polymorphisms in a replicated case-control study of Iranian preeclamptic women. Although the allele and genotype frequencies of seven assessed polymorphisms were not related to PE, four haplotypes of ACGACTT, GTCAGGA, ACGACGT, and GTGACTT were associated with the disease risk. Last but not least, it is important to study further to gain more insight into the role of *ERAP1* and *2* genes in the pathogenesis of preeclampsia; nevertheless, either determination of miscellaneous indices alongside with genetic analysis and investigation of various populations would be beneficial.

## Ethical considerations

Ethical issues (Including plagiarism, informed consent, misconduct, data fabrication and/or falsification, double publication and/or submission, redundancy, etc.) have been completely observed by the authors.
